# (*E*)-1-(6-Chloro-2-methyl-4-phenyl-3-quinol­yl)-3-(2-methoxy­phen­yl)prop-2-en-1-one

**DOI:** 10.1107/S1600536810000784

**Published:** 2010-01-13

**Authors:** Wan-Sin Loh, Hoong-Kun Fun, S. Sarveswari, V. Vijayakumar, B. Palakshi Reddy

**Affiliations:** a X-ray Crystallography Unit, School of Physics, Universiti Sains Malaysia, 11800 USM, Penang, Malaysia; b Organic Chemistry Division, School of Advanced Sciences, VIT University, Vellore 632 014, India

## Abstract

In the title compound, C_26_H_20_ClNO_2_, the quinoline ring system and the methoxy­phenyl ring form dihedral angles of 69.97 (6) and 22.10 (10)°, respectively, with the propenone linkage. The 4-phenyl ring substituent on the quinoline ring system is oriented at a dihedral angle of 66.47 (3)°. In the crystal, mol­ecules exist as C—H⋯O hydrogen-bonded dimers. The structure is further stabilized by C—H⋯π inter­actions.

## Related literature

For background details and the biological activity of quinolines, see: Michael (1997 ▶); Markees *et al.* (1970 ▶); Kalluraya & Sreenivasa (1998 ▶); Chen *et al.* (2001 ▶). For the biological activity of chalcones, see: Dimmock *et al.* (1999 ▶); Zi & Simoneau (2005 ▶). For related structures, see: Loh *et al.* (2009*a* ▶,*b* ▶). For bond-length data, see: Allen *et al.* (1987 ▶). For the stability of the temperature controller used in the data collection, see: Cosier & Glazer (1986 ▶).
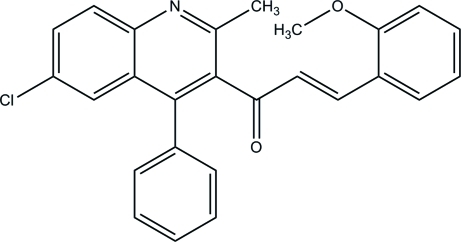


## Experimental

### 

#### Crystal data

C_26_H_20_ClNO_2_
*M* *_r_* = 413.88
Monoclinic, 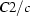

*a* = 15.1154 (2) Å
*b* = 15.4655 (2) Å
*c* = 17.2400 (2) Å
β = 104.418 (1)°
*V* = 3903.22 (9) Å^3^
*Z* = 8
Mo *K*α radiation
μ = 0.22 mm^−1^
*T* = 100 K
0.39 × 0.25 × 0.19 mm

#### Data collection

Bruker SMART APEXII CCD area-detector diffractometer
Absorption correction: multi-scan (*SADABS*; Bruker, 2009 ▶) *T*_min_ = 0.919, *T*_max_ = 0.960
30753 measured reflections
8197 independent reflections
5864 reflections with *I* > 2σ(*I*)
*R*_int_ = 0.039

#### Refinement

*R*[*F* ^2^ > 2σ(*F* ^2^)] = 0.053
*wR*(*F* ^2^) = 0.136
*S* = 1.06
8197 reflections
273 parameters
H-atom parameters constrained
Δρ_max_ = 0.54 e Å^−3^
Δρ_min_ = −0.31 e Å^−3^

### 

Data collection: *APEX2* (Bruker, 2009 ▶); cell refinement: *SAINT* (Bruker, 2009 ▶); data reduction: *SAINT*; program(s) used to solve structure: *SHELXTL* (Sheldrick, 2008 ▶); program(s) used to refine structure: *SHELXTL*; molecular graphics: *SHELXTL*; software used to prepare material for publication: *SHELXTL* and *PLATON* (Spek, 2009 ▶).

## Supplementary Material

Crystal structure: contains datablocks global, I. DOI: 10.1107/S1600536810000784/ci5015sup1.cif

Structure factors: contains datablocks I. DOI: 10.1107/S1600536810000784/ci5015Isup2.hkl

Additional supplementary materials:  crystallographic information; 3D view; checkCIF report

## Figures and Tables

**Table 1:** Hydrogen-bond geometry (Å, °) *Cg*1 and *Cg*2 are the centroids of the C2–C7 and N1/C1/C2/C7–C9 rings, respectively.

*D*—H⋯*A*	*D*—H	H⋯*A*	*D*⋯*A*	*D*—H⋯*A*
C12—H12*A*⋯O1^i^	0.93	2.59	3.2963 (16)	133
C17—H17*A*⋯*Cg*1^ii^	0.93	2.96	3.6617 (14)	134
C20—H20*A*⋯*Cg*2^ii^	0.93	2.85	3.6353 (14)	143

Symmetry codes: (i) 
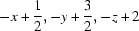
; (ii) 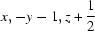.

**Fig. 1.**
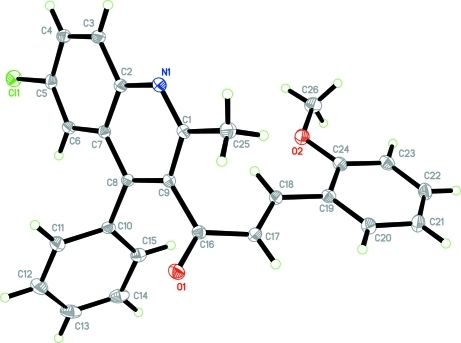
The molecular structure of the title compound, showing 50% probability displacement ellipsoids and the atom-numbering scheme.

**Fig. 2.**
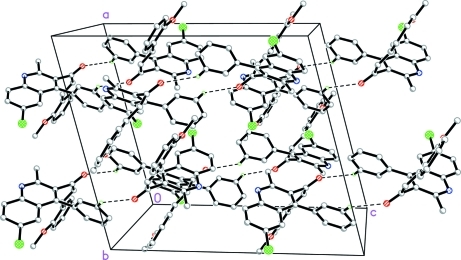
The crystal packing of the title compound, viewed approximately along the b axis, showing the dimers. H atoms not involved in the intermolecular interactions (dashed lines) have been omitted for clarity.

## supplementary crystallographic information

### Comment

Quinoline and its derivatives are very important compounds because of their wide occurrence in natural products (Michael, 1997) and biologically active compounds (Markees *et al.*, 1970). A large variety of quinolines have interesting physiological activities and found attractive applications as pharmaceuticals, agrochemicals and as synthetic building blocks (Kalluraya & Sreenivasa, 1998; Chen *et al.*, 2001). The chalcones are open chain flavonoids, possessing a variety of biological activities, including antioxidant, anti-inflammation, antimicrobial, antiprotozoal, antiulcer, as well as other activities (Dimmock *et al.*, 1999). More importantly, chalcones have shown several anticancer activities as inhibitors of cancer cell proliferation, carcinogenesis and metastasis (Zi & Simoneau, 2005).

In the molecule of the title compound (Fig. 1), the quinoline ring system (C1–C9/N1) is approximately planar with a maximum deviation of 0.065 (1) Å for atom C9. The mean plane of the quinoline ring system forms a dihedral angle of 66.47 (3)° with the C10-C15 phenyl ring. The C1–C9/N1 and C19-C24 planes form dihedral angles of 69.97 (6) and 22.10 (10)°, respectively, with the O1/C16-C18 plane. Bond lengths (Allen *et al.*, 1987) and angles are within the normal range and are comparable to closely related structures (Loh *et al.*, 2009*a*; Loh *et al.*, 2009*b*).

In the crystal (Fig. 2), pairs of neighbouring molecules are arranged into dimers by pairs of intermolecular C12—H12A···O1 hydrogen bonds. The crystal structure is further stabilized by C—H···π interactions (Table 1), involving C2–C7 (centroid *Cg*1) and N1/C1/C2/C7-C9 (centroid *Cg*2) rings.

### Experimental

A mixture of 3-acetyl-6-chloro-2-methyl-4-phenylquinoline (2.95 g, 0.01 mol), 2-methoxybenzaldehyde (1.36 g, 0.01 mol) and a catalytic amount of KOH in distilled ethanol was stirred for 12 h. The resulting mixture was concentrated to remove the ethanol and then poured onto ice and neutralized with diluted acetic acid. The resultant solid was filtered, dried and purified by column chromatography using a 1:1 mixture of ethyl acetate and petroleum ether (m.p. 403–405 K, yield: 68%).

### Refinement

H atoms were positioned geometrically [C–H = 0.93 or 0.96 Å] and were refined using a riding model, with *U*_iso_(H) = 1.2-1.5*U*_eq_(C). A rotating group model was applied to the methyl groups.

### Crystal data

**Table:**

C_26_H_20_ClNO_2_	*F*(000) = 1728
*M**_r_* = 413.88	*D*_x_ = 1.409 Mg m^−^^3^
Monoclinic, *C*2/*c*	Mo *K*α radiation, λ = 0.71073 Å
Hall symbol: -C 2yc	Cell parameters from 7486 reflections
*a* = 15.1154 (2) Å	θ = 2.4–31.5°
*b* = 15.4655 (2) Å	µ = 0.22 mm^−^^1^
*c* = 17.2400 (2) Å	*T* = 100 K
β = 104.418 (1)°	Block, yellow
*V* = 3903.22 (9) Å^3^	0.39 × 0.25 × 0.19 mm
*Z* = 8	

### Data collection

**Table:**

Bruker SMART APEXII CCD area-detector diffractometer	8197 independent reflections
Radiation source: fine-focus sealed tube	5864 reflections with *I* > 2σ(*I*)
graphite	*R*_int_ = 0.039
φ and ω scans	θ_max_ = 34.5°, θ_min_ = 2.4°
Absorption correction: multi-scan (*SADABS*; Bruker, 2009)	*h* = −20→24
*T*_min_ = 0.919, *T*_max_ = 0.960	*k* = −24→19
30753 measured reflections	*l* = −27→27

### Refinement

**Table:**

Refinement on *F*^2^	Primary atom site location: structure-invariant direct methods
Least-squares matrix: full	Secondary atom site location: difference Fourier map
*R*[*F*^2^ > 2σ(*F*^2^)] = 0.053	Hydrogen site location: inferred from neighbouring sites
*wR*(*F*^2^) = 0.136	H-atom parameters constrained
*S* = 1.06	*w* = 1/[σ^2^(*F*_o_^2^) + (0.0625*P*)^2^ + 0.7227*P*] where *P* = (*F*_o_^2^ + 2*F*_c_^2^)/3
8197 reflections	(Δ/σ)_max_ = 0.001
273 parameters	Δρ_max_ = 0.54 e Å^−^^3^
0 restraints	Δρ_min_ = −0.31 e Å^−^^3^

### Special details

**Table:**

Experimental. The crystal was placed in the cold stream of an Oxford Cyrosystems Cobra open-flow nitrogen cryostat (Cosier & Glazer, 1986) operating at 100.0 (1) K.
Geometry. All e.s.d.'s (except the e.s.d. in the dihedral angle between two l.s. planes) are estimated using the full covariance matrix. The cell e.s.d.'s are taken into account individually in the estimation of e.s.d.'s in distances, angles and torsion angles; correlations between e.s.d.'s in cell parameters are only used when they are defined by crystal symmetry. An approximate (isotropic) treatment of cell e.s.d.'s is used for estimating e.s.d.'s involving l.s. planes.
Refinement. Refinement of *F*^2^ against ALL reflections. The weighted *R*-factor *wR* and goodness of fit *S* are based on *F*^2^, conventional *R*-factors *R* are based on *F*, with *F* set to zero for negative *F*^2^. The threshold expression of *F*^2^ > σ(*F*^2^) is used only for calculating *R*-factors(gt) *etc*. and is not relevant to the choice of reflections for refinement. *R*-factors based on *F*^2^ are statistically about twice as large as those based on *F*, and *R*- factors based on ALL data will be even larger.

### Fractional atomic coordinates and isotropic or equivalent isotropic displacement parameters (Å^2^)

**Table:**

	*x*	*y*	*z*	*U*_iso_*/*U*_eq_	
Cl1	0.02177 (2)	1.01758 (2)	0.635395 (19)	0.01901 (8)	
O1	0.27571 (6)	0.58953 (6)	0.86651 (5)	0.0176 (2)	
O2	−0.04599 (6)	0.49228 (6)	0.58651 (5)	0.0182 (2)	
N1	0.23373 (7)	0.70354 (7)	0.61280 (6)	0.0132 (2)	
C1	0.24441 (8)	0.64331 (8)	0.66867 (7)	0.0119 (2)	
C2	0.18686 (8)	0.77723 (8)	0.62237 (7)	0.0118 (2)	
C3	0.17164 (9)	0.83936 (8)	0.55976 (7)	0.0140 (2)	
H3A	0.1948	0.8299	0.5153	0.017*	
C4	0.12304 (9)	0.91308 (8)	0.56457 (7)	0.0147 (2)	
H4A	0.1130	0.9538	0.5236	0.018*	
C5	0.08838 (8)	0.92650 (8)	0.63233 (7)	0.0137 (2)	
C6	0.10437 (8)	0.87006 (8)	0.69522 (7)	0.0130 (2)	
H6A	0.0826	0.8818	0.7400	0.016*	
C7	0.15466 (8)	0.79326 (8)	0.69143 (7)	0.0114 (2)	
C8	0.17037 (8)	0.72900 (8)	0.75300 (7)	0.0113 (2)	
C9	0.21100 (8)	0.65250 (8)	0.73916 (7)	0.0117 (2)	
C10	0.14444 (8)	0.74686 (8)	0.82932 (7)	0.0126 (2)	
C11	0.18819 (9)	0.81414 (9)	0.87804 (8)	0.0184 (3)	
H11A	0.2323	0.8468	0.8622	0.022*	
C12	0.16655 (11)	0.83282 (10)	0.94971 (8)	0.0234 (3)	
H12A	0.1966	0.8774	0.9819	0.028*	
C13	0.10036 (10)	0.78525 (10)	0.97343 (8)	0.0236 (3)	
H13A	0.0853	0.7983	1.0212	0.028*	
C14	0.05658 (9)	0.71812 (10)	0.92586 (8)	0.0213 (3)	
H14A	0.0122	0.6860	0.9419	0.026*	
C15	0.07867 (9)	0.69836 (9)	0.85395 (7)	0.0166 (2)	
H15A	0.0495	0.6528	0.8225	0.020*	
C16	0.22442 (8)	0.57996 (8)	0.79974 (7)	0.0125 (2)	
C17	0.17554 (9)	0.49843 (8)	0.77583 (7)	0.0144 (2)	
H17A	0.1908	0.4509	0.8095	0.017*	
C18	0.10990 (9)	0.48907 (8)	0.70786 (7)	0.0137 (2)	
H18A	0.0929	0.5384	0.6769	0.016*	
C19	0.06258 (8)	0.40889 (8)	0.67781 (7)	0.0135 (2)	
C20	0.09598 (9)	0.32817 (8)	0.70747 (8)	0.0161 (2)	
H20A	0.1493	0.3253	0.7484	0.019*	
C21	0.05186 (9)	0.25228 (9)	0.67757 (8)	0.0186 (3)	
H21A	0.0758	0.1990	0.6974	0.022*	
C22	−0.02865 (9)	0.25684 (9)	0.61751 (8)	0.0193 (3)	
H22A	−0.0595	0.2062	0.5983	0.023*	
C23	−0.06371 (9)	0.33563 (9)	0.58583 (8)	0.0171 (3)	
H23A	−0.1175	0.3377	0.5454	0.021*	
C24	−0.01789 (9)	0.41163 (8)	0.61490 (7)	0.0148 (2)	
C25	0.29908 (9)	0.56529 (8)	0.65696 (8)	0.0170 (2)	
H25A	0.3273	0.5758	0.6137	0.026*	
H25B	0.2594	0.5160	0.6445	0.026*	
H25C	0.3454	0.5542	0.7052	0.026*	
C26	−0.12419 (10)	0.49727 (10)	0.51992 (8)	0.0213 (3)	
H26A	−0.1348	0.5564	0.5034	0.032*	
H26B	−0.1767	0.4752	0.5353	0.032*	
H26C	−0.1136	0.4636	0.4763	0.032*	

### Atomic displacement parameters (Å^2^)

**Table:**

	*U*^11^	*U*^22^	*U*^33^	*U*^12^	*U*^13^	*U*^23^
Cl1	0.01956 (16)	0.01561 (16)	0.02069 (15)	0.00653 (12)	0.00279 (12)	0.00098 (11)
O1	0.0204 (5)	0.0172 (5)	0.0129 (4)	0.0012 (4)	−0.0004 (3)	−0.0009 (3)
O2	0.0176 (5)	0.0162 (5)	0.0172 (4)	0.0006 (4)	−0.0022 (4)	0.0005 (3)
N1	0.0144 (5)	0.0130 (5)	0.0124 (4)	0.0001 (4)	0.0039 (4)	−0.0009 (4)
C1	0.0120 (5)	0.0122 (6)	0.0117 (5)	−0.0003 (4)	0.0030 (4)	−0.0017 (4)
C2	0.0109 (5)	0.0123 (5)	0.0119 (5)	−0.0004 (4)	0.0026 (4)	−0.0004 (4)
C3	0.0148 (6)	0.0156 (6)	0.0112 (5)	−0.0014 (4)	0.0025 (4)	0.0004 (4)
C4	0.0158 (6)	0.0147 (6)	0.0125 (5)	−0.0006 (5)	0.0013 (4)	0.0014 (4)
C5	0.0130 (6)	0.0110 (5)	0.0155 (5)	0.0003 (4)	0.0008 (4)	−0.0005 (4)
C6	0.0139 (5)	0.0135 (6)	0.0118 (5)	0.0002 (4)	0.0034 (4)	−0.0010 (4)
C7	0.0108 (5)	0.0120 (6)	0.0111 (5)	−0.0003 (4)	0.0021 (4)	−0.0001 (4)
C8	0.0113 (5)	0.0121 (5)	0.0106 (5)	−0.0017 (4)	0.0028 (4)	−0.0001 (4)
C9	0.0120 (5)	0.0116 (5)	0.0111 (5)	−0.0007 (4)	0.0022 (4)	−0.0005 (4)
C10	0.0152 (6)	0.0121 (5)	0.0107 (5)	0.0031 (4)	0.0038 (4)	0.0011 (4)
C11	0.0228 (7)	0.0180 (6)	0.0154 (5)	−0.0025 (5)	0.0069 (5)	−0.0022 (5)
C12	0.0329 (8)	0.0227 (7)	0.0153 (6)	0.0010 (6)	0.0073 (6)	−0.0046 (5)
C13	0.0293 (8)	0.0305 (8)	0.0135 (5)	0.0091 (6)	0.0103 (5)	0.0023 (5)
C14	0.0206 (7)	0.0282 (8)	0.0186 (6)	0.0042 (6)	0.0116 (5)	0.0059 (5)
C15	0.0170 (6)	0.0179 (6)	0.0157 (5)	0.0009 (5)	0.0054 (5)	0.0019 (5)
C16	0.0132 (5)	0.0132 (6)	0.0116 (5)	0.0015 (4)	0.0042 (4)	−0.0001 (4)
C17	0.0180 (6)	0.0119 (6)	0.0139 (5)	0.0003 (4)	0.0052 (5)	0.0012 (4)
C18	0.0152 (6)	0.0129 (6)	0.0136 (5)	−0.0001 (4)	0.0047 (4)	−0.0003 (4)
C19	0.0126 (5)	0.0155 (6)	0.0132 (5)	−0.0009 (4)	0.0047 (4)	−0.0006 (4)
C20	0.0140 (6)	0.0166 (6)	0.0170 (6)	0.0000 (5)	0.0026 (5)	−0.0017 (5)
C21	0.0200 (6)	0.0130 (6)	0.0218 (6)	0.0005 (5)	0.0036 (5)	−0.0012 (5)
C22	0.0215 (7)	0.0163 (6)	0.0201 (6)	−0.0037 (5)	0.0052 (5)	−0.0041 (5)
C23	0.0156 (6)	0.0204 (6)	0.0150 (5)	−0.0024 (5)	0.0032 (5)	−0.0031 (5)
C24	0.0144 (6)	0.0167 (6)	0.0140 (5)	0.0004 (5)	0.0048 (4)	−0.0016 (4)
C25	0.0193 (6)	0.0160 (6)	0.0168 (6)	0.0046 (5)	0.0065 (5)	0.0005 (5)
C26	0.0179 (7)	0.0245 (7)	0.0178 (6)	0.0014 (5)	−0.0025 (5)	0.0019 (5)

### Geometric parameters (Å, °)

**Table:**

Cl1—C5	1.7397 (13)	C13—C14	1.385 (2)
O1—C16	1.2262 (14)	C13—H13A	0.93
O2—C24	1.3683 (15)	C14—C15	1.3956 (17)
O2—C26	1.4302 (16)	C14—H14A	0.93
N1—C1	1.3207 (15)	C15—H15A	0.93
N1—C2	1.3733 (15)	C16—C17	1.4678 (18)
C1—C9	1.4344 (16)	C17—C18	1.3414 (17)
C1—C25	1.5042 (17)	C17—H17A	0.93
C2—C7	1.4158 (16)	C18—C19	1.4606 (18)
C2—C3	1.4205 (17)	C18—H18A	0.93
C3—C4	1.3700 (18)	C19—C20	1.3951 (18)
C3—H3A	0.93	C19—C24	1.4144 (18)
C4—C5	1.4098 (17)	C20—C21	1.3845 (18)
C4—H4A	0.93	C20—H20A	0.93
C5—C6	1.3657 (16)	C21—C22	1.3895 (18)
C6—C7	1.4204 (17)	C21—H21A	0.93
C6—H6A	0.93	C22—C23	1.3855 (19)
C7—C8	1.4302 (16)	C22—H22A	0.93
C8—C9	1.3808 (17)	C23—C24	1.3926 (18)
C8—C10	1.4893 (16)	C23—H23A	0.93
C9—C16	1.5116 (17)	C25—H25A	0.96
C10—C15	1.3934 (18)	C25—H25B	0.96
C10—C11	1.3960 (18)	C25—H25C	0.96
C11—C12	1.3851 (18)	C26—H26A	0.96
C11—H11A	0.93	C26—H26B	0.96
C12—C13	1.383 (2)	C26—H26C	0.96
C12—H12A	0.93		
			
C24—O2—C26	117.21 (10)	C15—C14—H14A	119.8
C1—N1—C2	118.08 (10)	C10—C15—C14	119.97 (12)
N1—C1—C9	122.90 (11)	C10—C15—H15A	120.0
N1—C1—C25	116.07 (10)	C14—C15—H15A	120.0
C9—C1—C25	120.94 (10)	O1—C16—C17	121.58 (11)
N1—C2—C7	122.82 (11)	O1—C16—C9	120.26 (11)
N1—C2—C3	117.44 (10)	C17—C16—C9	118.16 (10)
C7—C2—C3	119.74 (11)	C18—C17—C16	123.30 (11)
C4—C3—C2	120.32 (11)	C18—C17—H17A	118.3
C4—C3—H3A	119.8	C16—C17—H17A	118.3
C2—C3—H3A	119.8	C17—C18—C19	126.29 (12)
C3—C4—C5	119.23 (11)	C17—C18—H18A	116.9
C3—C4—H4A	120.4	C19—C18—H18A	116.9
C5—C4—H4A	120.4	C20—C19—C24	118.13 (11)
C6—C5—C4	122.34 (11)	C20—C19—C18	121.94 (11)
C6—C5—Cl1	118.87 (10)	C24—C19—C18	119.89 (11)
C4—C5—Cl1	118.79 (9)	C21—C20—C19	121.69 (12)
C5—C6—C7	119.27 (11)	C21—C20—H20A	119.2
C5—C6—H6A	120.4	C19—C20—H20A	119.2
C7—C6—H6A	120.4	C20—C21—C22	119.06 (12)
C2—C7—C6	119.02 (10)	C20—C21—H21A	120.5
C2—C7—C8	118.30 (11)	C22—C21—H21A	120.5
C6—C7—C8	122.60 (11)	C23—C22—C21	121.09 (12)
C9—C8—C7	117.77 (10)	C23—C22—H22A	119.5
C9—C8—C10	122.53 (10)	C21—C22—H22A	119.5
C7—C8—C10	119.68 (11)	C22—C23—C24	119.56 (12)
C8—C9—C1	119.79 (10)	C22—C23—H23A	120.2
C8—C9—C16	120.36 (10)	C24—C23—H23A	120.2
C1—C9—C16	119.79 (10)	O2—C24—C23	123.95 (11)
C15—C10—C11	119.01 (11)	O2—C24—C19	115.63 (11)
C15—C10—C8	122.28 (11)	C23—C24—C19	120.42 (12)
C11—C10—C8	118.71 (11)	C1—C25—H25A	109.5
C12—C11—C10	120.69 (13)	C1—C25—H25B	109.5
C12—C11—H11A	119.7	H25A—C25—H25B	109.5
C10—C11—H11A	119.7	C1—C25—H25C	109.5
C13—C12—C11	120.11 (13)	H25A—C25—H25C	109.5
C13—C12—H12A	119.9	H25B—C25—H25C	109.5
C11—C12—H12A	119.9	O2—C26—H26A	109.5
C12—C13—C14	119.85 (12)	O2—C26—H26B	109.5
C12—C13—H13A	120.1	H26A—C26—H26B	109.5
C14—C13—H13A	120.1	O2—C26—H26C	109.5
C13—C14—C15	120.36 (13)	H26A—C26—H26C	109.5
C13—C14—H14A	119.8	H26B—C26—H26C	109.5
			
C2—N1—C1—C9	−1.36 (17)	C7—C8—C10—C11	−63.24 (16)
C2—N1—C1—C25	−177.88 (10)	C15—C10—C11—C12	−0.21 (19)
C1—N1—C2—C7	3.89 (17)	C8—C10—C11—C12	−179.71 (12)
C1—N1—C2—C3	−176.69 (11)	C10—C11—C12—C13	−0.6 (2)
N1—C2—C3—C4	178.10 (11)	C11—C12—C13—C14	0.9 (2)
C7—C2—C3—C4	−2.46 (18)	C12—C13—C14—C15	−0.2 (2)
C2—C3—C4—C5	0.00 (18)	C11—C10—C15—C14	0.85 (19)
C3—C4—C5—C6	2.54 (19)	C8—C10—C15—C14	−179.67 (11)
C3—C4—C5—Cl1	−176.70 (10)	C13—C14—C15—C10	−0.6 (2)
C4—C5—C6—C7	−2.50 (18)	C8—C9—C16—O1	−64.94 (16)
Cl1—C5—C6—C7	176.75 (9)	C1—C9—C16—O1	112.20 (13)
N1—C2—C7—C6	−178.11 (11)	C8—C9—C16—C17	115.85 (13)
C3—C2—C7—C6	2.48 (17)	C1—C9—C16—C17	−67.02 (15)
N1—C2—C7—C8	−1.18 (17)	O1—C16—C17—C18	170.75 (12)
C3—C2—C7—C8	179.42 (11)	C9—C16—C17—C18	−10.04 (18)
C5—C6—C7—C2	−0.04 (17)	C16—C17—C18—C19	175.84 (12)
C5—C6—C7—C8	−176.84 (11)	C17—C18—C19—C20	−15.8 (2)
C2—C7—C8—C9	−4.05 (17)	C17—C18—C19—C24	166.47 (12)
C6—C7—C8—C9	172.77 (11)	C24—C19—C20—C21	−0.80 (18)
C2—C7—C8—C10	174.87 (11)	C18—C19—C20—C21	−178.61 (12)
C6—C7—C8—C10	−8.31 (17)	C19—C20—C21—C22	−1.21 (19)
C7—C8—C9—C1	6.47 (17)	C20—C21—C22—C23	1.8 (2)
C10—C8—C9—C1	−172.42 (11)	C21—C22—C23—C24	−0.39 (19)
C7—C8—C9—C16	−176.40 (10)	C26—O2—C24—C23	−3.77 (18)
C10—C8—C9—C16	4.71 (18)	C26—O2—C24—C19	176.59 (11)
N1—C1—C9—C8	−3.94 (18)	C22—C23—C24—O2	178.69 (12)
C25—C1—C9—C8	172.42 (11)	C22—C23—C24—C19	−1.69 (19)
N1—C1—C9—C16	178.91 (11)	C20—C19—C24—O2	−178.09 (11)
C25—C1—C9—C16	−4.73 (17)	C18—C19—C24—O2	−0.24 (17)
C9—C8—C10—C15	−63.86 (17)	C20—C19—C24—C23	2.26 (18)
C7—C8—C10—C15	117.28 (13)	C18—C19—C24—C23	−179.89 (11)
C9—C8—C10—C11	115.63 (14)		

### Hydrogen-bond geometry (Å, °)

**Table:**

*D*—H···*A*	*D*—H	H···*A*	*D*···*A*	*D*—H···*A*
C12—H12A···O1^i^	0.93	2.59	3.2963 (16)	133
C17—H17A···Cg1^ii^	0.93	2.96	3.6617 (14)	134
C20—H20A···Cg2^ii^	0.93	2.85	3.6353 (14)	143

Symmetry codes: (i) −*x*+1/2, −*y*+3/2, −*z*+2; (ii) *x*, −*y*−1, *z*+1/2.
